# SDPR Inhibits TGF-β Induced Cancer Metastasis Through Fatty Acid Oxidation Regulation in Gastric Cancer

**DOI:** 10.7150/ijbs.83012

**Published:** 2023-06-04

**Authors:** Xiaoyue Li, Jing Luo, Kelin Mou, Lin Peng, Huan Zhou, Yulin Lei, Huan Wang, Zhengfei Zhao, Jianmei Wang, Jianhua Wu, Runlan Wan, Sheng Lin, Li Xiang, Yuhao Luo

**Affiliations:** 1 Department of Oncology, The Affiliated Hospital of Southwest Medical University, Sichuan, Luzhou, 644000, China.; 2 Department of Cardiovascular Medicine, The Affiliated Hospital of Southwest Medical University, Sichuan, Luzhou, China.; 3 Department of Bone and Joint, The Affiliated Hospital of Southwest Medical University, Luzhou, China.; 4 Department of Gastrointestinal Surgery, The Affiliated Hospital of Southwest Medical University, Sichuan, Luzhou, China.; 5 Department of Pathology, The Affiliated Hospital of Southwest Medical University, Luzhou, China.; 6 Department of Oncology, Nanfang Hospital, Southern Medical University, Guangzhou, 510000, China.; 7 Nuclear Medicine and Molecular Imaging Key Laboratory of Sichuan Province, Luzhou, China.

**Keywords:** TGF-β, SDPR, CPT1A, fatty acid oxidation, gastric cancer

## Abstract

Our previous studies have confirmed that transforming growth factor-β (TGF-β) plays an important role in tumor metastasis, and the serum deprivation protein response (SDPR) is a potential downstream target of TGF-β. However, the role and mechanism of SDPR in gastric cancer are still unclear. We performed gene microarray, bioinformation analysis, combined with *in vivo* and *in vitro* experimental verification, we identified that SDPR is significantly downregulated in gastric cancer, and participates in TGF-β-mediated tumour metastasis. Mechanically, SDPR interacts with extracellular signal-regulated kinase (ERK) and inhibits fatty acid metabolism key gene Carnitine palmitoyl transferase 1A (CPT1A) at transcriptional level by supressing ERK/PPAR pathway. Our findings suggest that the TGF-β/SDPR/CPT1A axis play an important role in the fatty acid oxidation of gastric cancer, and provides a new insight into the crosstalk of tumour microenvironments and metabolism reprogramming and suggest that strategies to intervene the fatty acid metabolism may therapy gastric cancer metastasis.

## Introduction

Gastric cancer is a common digestive tumour that seriously threatens human life and health and is the second leading cause of cancer-related deaths worldwide 1-3. In China, its morbidity and mortality rates can be as high as 28.68/100,000 and 20.87/100,000, respectively, ranking third among malignant tumours 4. Invasion and metastasis of tumours often require sufficient energy supply, and the production of energy is closely related to metabolism 5. Tumour invasion and metastasis are important reasons for poor patient prognosis. Therefore, exploring new anti-tumour metastasis strategies and finding new therapeutic targets to effectively curb the metastasis of gastric cancer remains a global concern.

TGF-β is an important regulator of the tumour microenvironment. Tumour metastasis is an important process in the interaction between tumour cells and their microenvironment 6. In colorectal cancer, TGF-β activates the classical Smad2 signalling pathway and promotes epithelial-mesenchymal transition (EMT) and tumour metastasis by modulating the expression of lim and sh3 protein 1 (LASP1) and noncoding RNA miR-187 7, 8. We also found that TGF-β promoted the expression of miR-577 at the transcriptional level through the activation of the NF-κB pathway by inhibiting SDPR, which ultimately promoted EMT and tumour stemness in gastric cancer 9, providing ideas and methods for the design and verification of this study. However, the downstream mechanisms of TGF-β regulation remain unclear.

SDPR, a member of the caveolin family, shows low expression in breast cancer and kidney cancer, is associated with a good prognosis of patients, and plays an important role as a tumour suppressor gene 10, 11. Interestingly, Tian et al. found that activation of SDPR could block the TGF-β pathway to inhibit breast cancer progression and EMT 12. Our previous study confirmed that miRNAs can enhance the TGF-β signalling pathway by targeting SDPR to form a positive feedback loop and ultimately promote the progression of gastric cancer 9. However, the mechanism by which SDPR mediates gastric cancer metastasis remains unclear.

Fatty acid oxidation is a process of lipid decomposition and is an important method by which malignant tumour cells obtain energy 13. In recent years, many studies reported that abnormal fatty acid oxidation plays a crucial role in the occurrence and development of breast, colorectal, and prostate cancer 14-16. Gatza et al. reported that CPT1A plays an important role in promoting cell growth and metastasis in breast and gastric cancers 17, 18. In contrast, CPT1 inhibits tumour tissue growth when downregulated in leukaemia and lymphoma 19. Therefore, targeting the key enzyme, CPT1A, in fatty acid oxidation may become a key method in regulating lipid metabolism. To date, relevant exploration of the regulatory mechanisms of lipid metabolism in gastric cancer is still very limited. Therefore, exploring the role of abnormal fatty acid oxidation in tumour biological behaviour and targeting its metabolic pathways may be a new strategy for curbing gastric cancer metastasis.

The purpose of this study was to identify the key role of TGF-β/SDPR/CPT1A signaling axis in gastric cancer metastasis, thus providing a new direction for the exploration of clinical therapeutic targets.

## Materials and Methods

### Cell lines

The human gastric cancer cell lines MGC803 and MKN45 used in this study were purchased from Guangzhou Xiaofan Technology and authenticated using the short tandem repeat (STR) method.

### Cell culture, transwell, wound-healing, immunohistochemistry (IHC), Haematoxylin and eosin (HE) staining

Cell culture, cell transfection, transwell, wound-healing, IHC, HE assays were performed according to previously described methods 9.

### Construction of siRNA and sh-RNA, and Cell Transfection

SDPR was overexpressed, knocked out and CPT1A knocked out by lentivirus (HanBio Technology, China). These assays were performed according to previously described methods 9. The siRNAs sequences were list at Supplementary Table S1.

### qRT-PCR and Western blot assays

These assays were performed according to previously described methods 9. All the primers designed for qPCR were listed in Supplementary Table S2. The details of antibodies for the western blot are listed in Supplementary Table S3.

### Cellular ATP detection

In this experiment, an ATP Assay Kit (S0026, Beyotime, Jiangsu, China) was used to detect the ATP level in cells, and the assay was carried out according to the manufacturer's instructions. The treated cells were inoculated into a six-well plate, and 200 µL lysis buffer was added to each well to lyse the cells. After lysis, the cells were centrifuged at 4 °C for 5 min and the supernatant was collected. The ATP detection working fluid was prepared according to the manufacturer's instructions. Subsequently, the samples to be tested were added, and chemiluminescence intensity was detected using a luminometer. Finally, the ATP concentration in the sample was calculated using a standard curve.

### Fatty acid oxidation (FAO) assay

First, according to the kit instructions, the Cell Mitochondria Isolation Kit (C3601, Beyotime, Jiangsu, China) was used to extract cell mitochondria, and then the fatty acid β oxidation rate of the treated cells was detected using a colorimetric detection kit (50679v. A). This mainly includes background control determination, sample determination, and calculation of the oxidation rate. Oxidation rate was calculated using the formula: [(sample reading - background reading) ×1 (system capacity; ml)]÷[0.5(sample protein volume; mg) ×105 (millimole absorbance coefficient) ×5 (reaction time; min)]= micromole ferricyanide reduction/min/mg.

### Chromatin immunoprecipitation (ChIP) assay

We performed ChIP assays using the Plus Enzymatic ChIP Kit (CST, Boston, MA, USA) to study the direct interactions between PPARα and CPT1A promoters according to the kit's reference guidelines. Briefly, the cells were processed and harvested, and for each immunoprecipitation reaction, the antibodies recommended by the protocol were added to each microcentrifuge tube, incubated overnight at 4°C, and then incubated with the supplied magnetic beads in rotation for 2 h at 4°C. Chromatin elution, reverse transfer linkage, and DNA purification were performed using the recommended sequences. The degree of enrichment was determined by real-time PCR. Each experiment was repeated three times.

### Dual luciferase assay

The PPARα sequence was cloned into the pcDNA3.1 plasmid vector. To generate the CPT1A promoter vector, we introduced a wild-type fragment containing the PPARα-binding site into the PGL4 luciferase reporter vector. The PPARα binding site mutant vector was constructed in Figure 4H. The PGL4-derived vector and PPARα plasmid were co-transfected into 293T, MGC803, and MKN45 cells using the Lipofectamine 2000 reagent (Invitrogen, Carlsbad, CA, USA). After 48 h, luciferase activity was measured using a dual luciferase reporter assay kit (CAT:11402ES60) according to the manufacturer's protocol.

### Gene microarray and bioinformatics

The cBioPortal web was used to analyse the coexpression genes of SDPR in The Cancer Genome Atlas (TCGA) STAD (gastric adenocarcinoma) database. The differentially expression genes between MGC803 cells and MGC803/SDPR cells were analysed, statistical significance was set at P < 0.001 and |log2 FC| >1, the differentially expressed genes were list at Supplemental Table S4. R package 20 was used to analyse the function and pathways of differentially expression genes. To identify transcription factors that regulate CPT1A, PROMO was used to analyse the promoter region-binding sites. In addition, the Kaplan-Meier Plotter was used to confirm the expression of SDPR and its prognostic role in gastric cancer.

### Tumour tissue samples

All tumour tissues were provided by the Tumour Tissue Bank of Nanfang Hospital and The Affiliated Hospital of Southwest Medical University. All specimens were attached to a confirmed pathological diagnosis. None of the patients had received any chemoradiotherapy or immunotherapy before surgery. All experiments were endorsed by the Ethics Committee of Southern Medical University and Southwest Medical University, and complied with the Declaration of Helsinki.

### Mouse xenograft model

For the *in vivo* metastasis experiment, all the experiments used male nude mice, 1*10^6^ cells were injected into the tail vein of nude mice. After 42 days, the mice were anaesthetised and intraperitoneal injection of fluorescein was used for *in vivo* imaging to detect systemic tumour metastasis. The endpoint was observed 60 days after injection or the natural death of mice. Lung tissue was fixed, metastatic tumours were detected by HE staining, and the expression of corresponding genes in different groups was detected by IHC. All animal experiments were approved by the Animal Ethics Committee of Southwest Medical University.

### Statistical analysis

All experiments were repeated three times. All statistical analyses were performed using SPSS v21.0 software and GraphPad Prism 8.0.1 software. A P value less than 0.05 was considered statistically significant.

## Results

### SDPR inhibits tumour metastasis *in vitro* and *in vivo*

**Figure 1:**
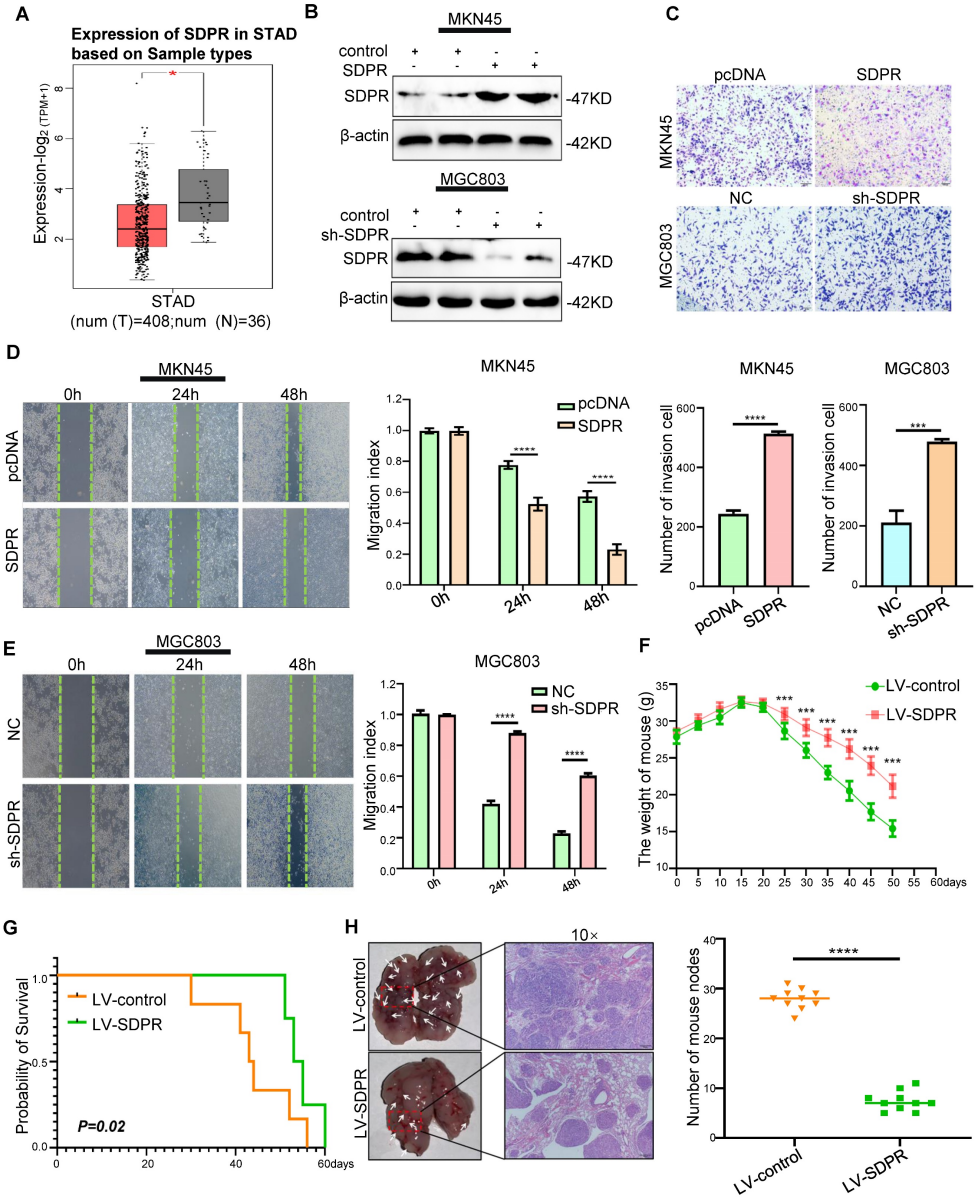
** SDPR inhibits tumour metastasis *in vitro* and vivo.** (A) The differential expression of SDPR in gastric cancer and normal tissue using TCGA microarray analysis. (B) Western blot was used to verify the efficiency of SDPR overexpression and knockdown, respectively. (C) Transwell chamber migration assay was used to detect the migration and invasion abilities of MKN45 and MGC803 cells after SDPR overexpression or knockdown. The bar graph below shows the number of cells that have invaded. (D-E) The wound healing assay was used to detect the migration ability of MKN45 (D) and MGC803(E). The bars on the right represent migration indices. (F) Weight change curves of mice injected with SDPR-overexpressing and control cells. (G) Kaplan-Meier survival curves for mice injected with SDPR-overexpressing cells and control cells. *P* = 0.02. (H) Comparison of lung metastases in mice injected with overexpressing and control cells. ***P < 0.001; **** P < 0.0001. These experiments were repeated three times.

We found low expression of SDPR in gastric cancer using TCGA microarray analysis (Figure 1A). We selected MGC803 (high SDPR expression) and MKN45 cells (low SDPR expression) for the experiments. *In vitro*, SDPR overexpression was performed on MKN45 cells and SDPR knockdown MGC803 cells. Western blot (Figure 1A, S1A) and qRT-PCR (Figure S1B) were used to determine the efficiency of the overexpression and knockout. The results showed that overexpression of SDPR significantly inhibited the invasion and migration of gastric cancer cells, while downregulation of SDPR resulted in the opposite results (Figure 1C-E). *In vivo*, compared to the control group, overexpression of SDPR significantly improved the survival and prognosis of nude mice (Figure 1F-G) and inhibited the formation of lung metastases (Figure 1H). HE staining of the tissue pathological sections from the model mice confirmed that SDPR had no significant effect on the heart, liver, spleen, or kidney (Figure S1C). The results of *in vivo* experiments further confirmed that SDPR can significantly inhibit the metastasis of gastric cancer.

### SDPR is involved in TGF-β-mediated gastric cancer metastasis

**Figure 2:**
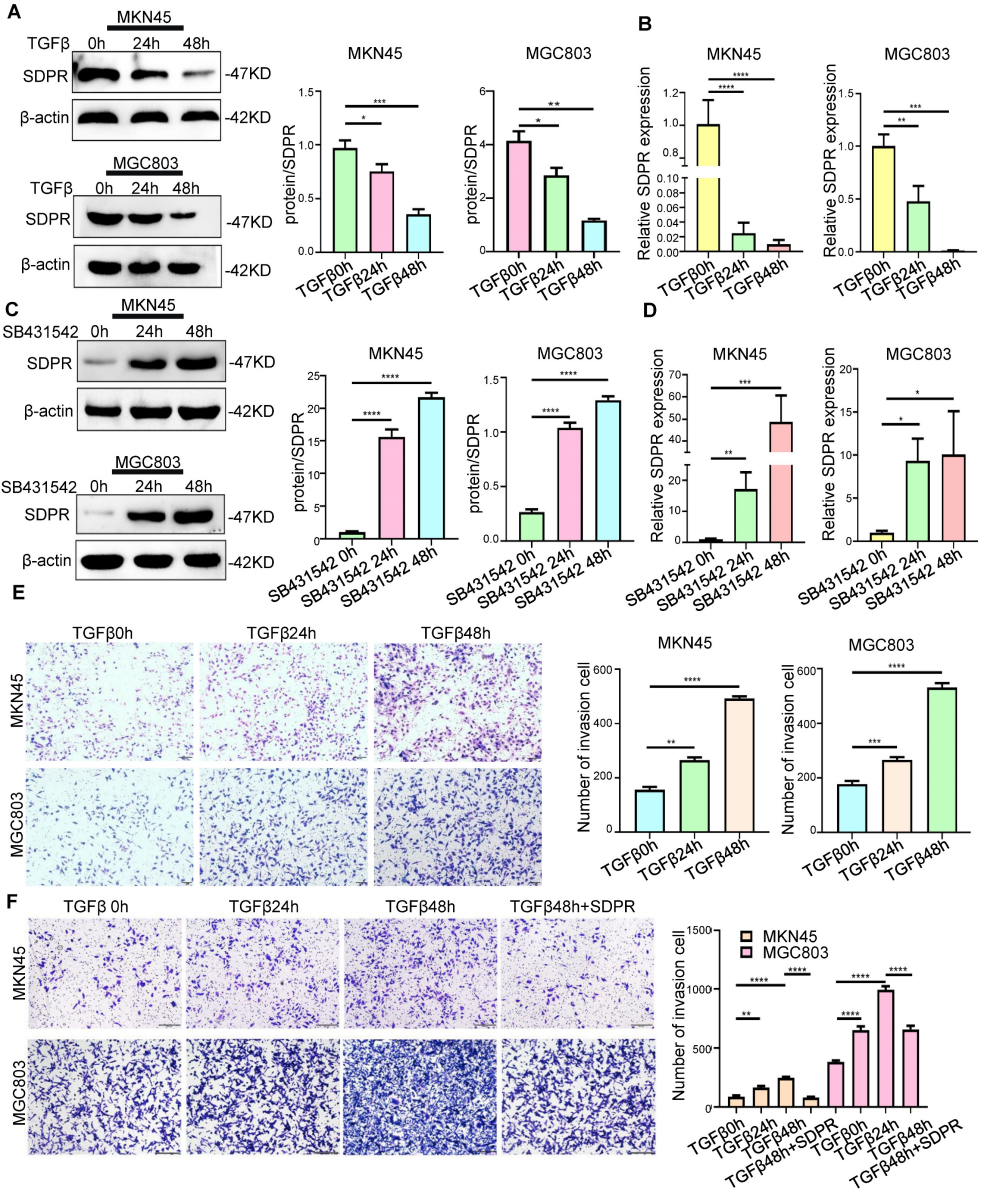
** SDPR is involved in TGF-β-mediated gastric cancer metastasis.** (A-B) Western blot and qRT-PCR were used to detect the changes of SDPR in MKN45 and MGC803 cells after 0, 24, and 48h of TGF-β treatment. (C-D) Western blot and qRT-PCR were used to detect the changes of SDPR in MKN45 and MGC803 cells treated with TGFβ inhibitor (SB431542) for 0,24, or 48h. (E) Transwell chamber migration assay was used to detect the migration and invasion ability of MKN45 and MGC803 cells treated with TGF-β for 0, 24, or 48h. Bars on the right represent the number of invaded cells. (F) Transwell recovery assay was used to detect the migration and invasion ability of MKN45 cells and MGC803 treated with TGF-β for 0, 24, or 48h and SDPR overexpression +TGF-β for 48h. The cells were counted under a microscope in five randomly selected fields. Magnification, 100×. ** P < 0.01; *** P < 0.001; **** P < 0.0001. These experiments were repeated three times.

In previous studies, we identified SDPR as a downstream target gene of TGF-β. We further confirmed the involvement of SDPR in TGF-β-mediated gastric cancer metastasis. The results of Western blot (Figure 2A) and qRT-PCR (Figure 2B) showed that adding TGF-β stimulating factor to MKN45 and MGC803 cells downregulated protein and RNA levels of SDPR in a time-dependent manner. In contrast, when TGF-β inhibitor (SB431542) was added to both cells, the opposite results were obtained (Figure 2C-D), further confirming that SDPR is a downstream target of TGF-β. We further explored the relevant role of TGF-β in gastric cancer to determine whether it mediates tumour metastasis. By adding TGF-β-stimulating factor to MKN45 and MGC803 cells, we found that TGF-β can promote cell invasion in a time-dependent manner (Figure 2E). In contrast, when TGF-β inhibitor was added to both cells, the opposite results were obtained (Figure S2A-B). To further clarify whether SDPR is involved in TGF-β-mediated gastric cancer metastasis, we performed a rescue experiment, and the results showed that the enhancement of cell invasion by TGF-β treatment was abrogated by the overexpression of SDPR (Figure 2F). In conclusion, we conclude that SDPR is involved in TGF-β-mediated gastric cancer metastasis, and TGF-β promotes gastric cancer metastasis by inhibiting the expression of SDPR.

### SDPR inhibits fatty acid oxidation

**Figure 3:**
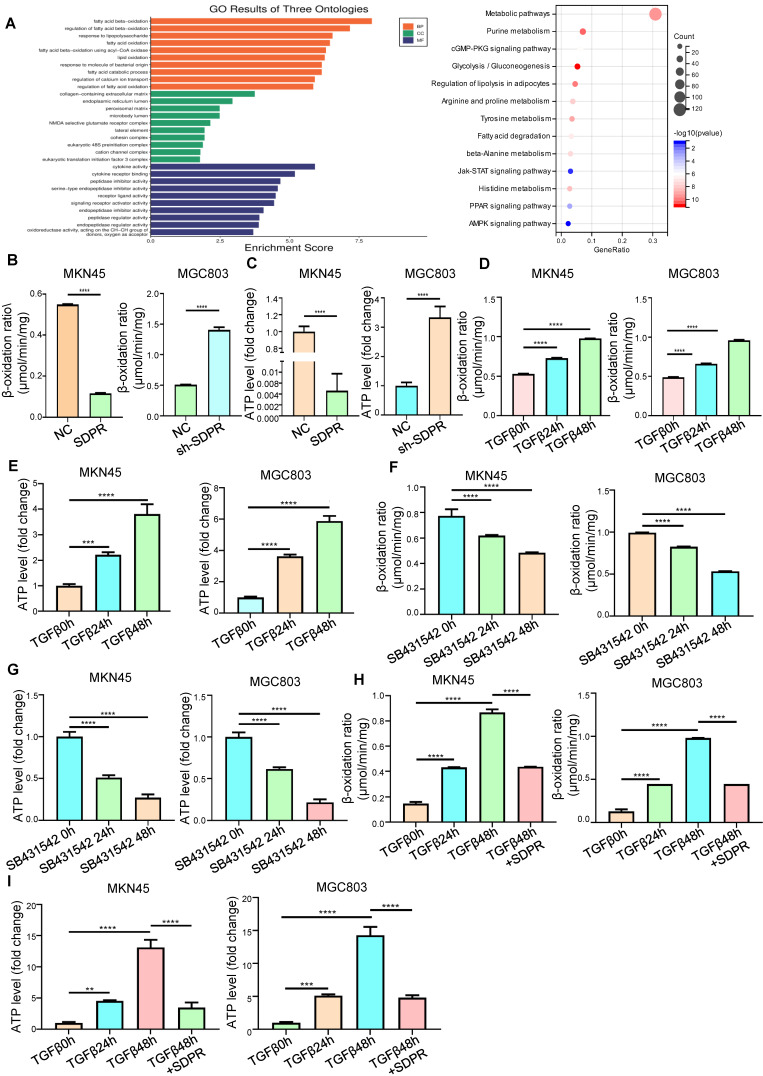
** SDPR inhibits fatty acid oxidation.** (A) Results of functional enrichment analysis of potential functions of SDPR by GO and KEGG databases. (B-C) FAO rate and ATP level of MKN45 and MGC803 cells after SDPR overexpression and knockdown. (D-E) FAO rate and ATP level of MKN45 and MGC803 cells treated with TGF-β for 0, 24, and 48h. (F-G) FAO rate and ATP level of MKN45 and MGC803 cells treated with TGF-β inhibitor (SB431542) for 0, 24, and 48h. (H-I) The recovery experiment showed the FAO rate and ATP level of MKN45 and MGC803 cells treated with TGF-β for 0, 24, or 48h and TGF-β 48h+ overexpression of SDPR. ** P < 0.01; *** P < 0.001; **** P < 0.0001. These experiments were repeated three times.

To further explore the molecular mechanism by which SDPR inhibits gastric cancer metastasis, we screened SDPR-related differential genes by high-throughput expression microarray profiling and performed GO analysis and KEGG enrichment of the differential genes. We found that SDPR may be related to fatty acid oxidation and PPAR pathway (Figure 3A). Therefore, we measured the FAO rate and ATP level in the MKN45 and MGC803 cells. The results showed that overexpression of SDPR significantly inhibited FAO. Conversely, knockout of SDPR can promote the FAO (Figure 3B-C). We further explored whether TGF-β, upstream of SDPR, regulates FAO. The results showed that in MKN45 and MGC803 cells, TGF-β promoted FAO rate and ATP level in a time-dependent manner (Figure 3D-E), whereas the TGF-β inhibitor had the opposite effect (Figure 3F-G). To further confirm that TGF-β upregulated the FAO rate and ATP level by inhibiting SDPR, we conducted a recovery experiment, which showed that SDPR restored the FAO rate and ATP level upregulated by TGF β (Figure 3H-I). The results showed that SDPR inhibited the oxidation of fatty acids, and TGF-β promoted the oxidation of fatty acids by inhibiting SDPR.

### SDPR regulates CPT1A

**Figure 4:**
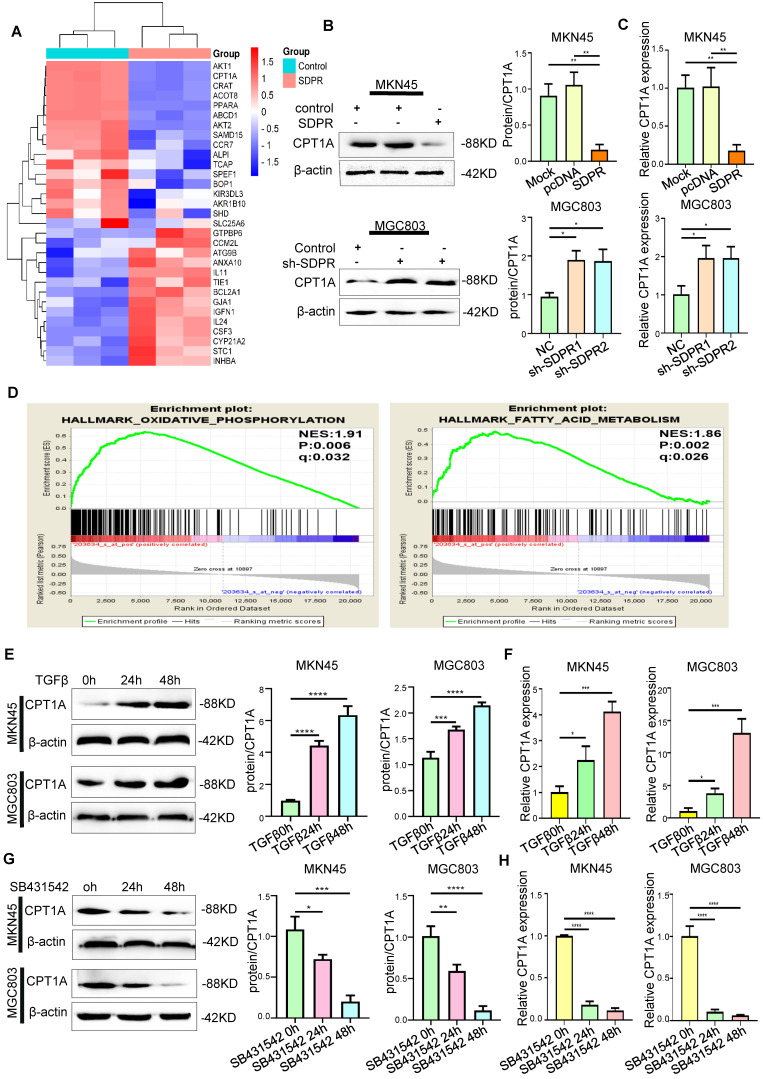
** SDPR regulates CPT1A.** (A) Heat map of overexpressed SDPR-related differential genes. (B-C) Western blot and qRT-PCR were used to detect the changes of CPT1A in MKN45 and MGC803 cells after SDPR overexpression and knockdown. (D) Gene function enrichment analysis of the GEO gastric cancer public database GSE57303 showed that CPT1A is associated with fatty acid metabolism. (E-F) Western blot and qRT-PCR were used to detect the changes of CPT1A in MKN45 and MGC803 cells treated with TGF-β for 0, 24, or 48h. (F-H) Western blot and qRT-PCR were used to detect the changes of CPT1A in MKN45 and MGC803 cells treated with TGFβ inhibitor (SB431542) for 0, 24, or 48h. ** P < 0.01; *** P < 0.001; **** P < 0.0001. These experiments were repeated three times.

We screened SDPR-related differential genes using a high-throughput expression profile microarray and found that upregulated SDPR could significantly inhibit CPT1A and PPARα (Figure 4A). The Western blot and qRT-PCR results showed that the upregulated SDPR significantly inhibited the expression of CPT1A at the protein and RNA levels; the opposite result was obtained by downregulating SDPR (Figure 4B-C). Interestingly, gene function enrichment analysis of the GEO gastric cancer public database GSE57303 showed that CPT1A is associated with fatty acid metabolism and FAO (Figure 4D). It was further confirmed that SDPR may regulate lipid metabolism by inhibiting CPT1A. Previous experiments demonstrated that TGF-β can inhibit the expression of SDPR. Therefore, we further confirmed that TGF-β can upregulate CPT1A expression in a time-dependent manner by Western blot and qRT-PCR assays (Figure 4E-F), the opposite result was obtained with TGF-β inhibitor (Figure 4G-H) in MKN45 and MGC803 cells. The above biological information and experimental results confirm that SDPR regulates the expression of CPT1A, but the specific regulatory mechanism is still unclear.

**Figure 5:**
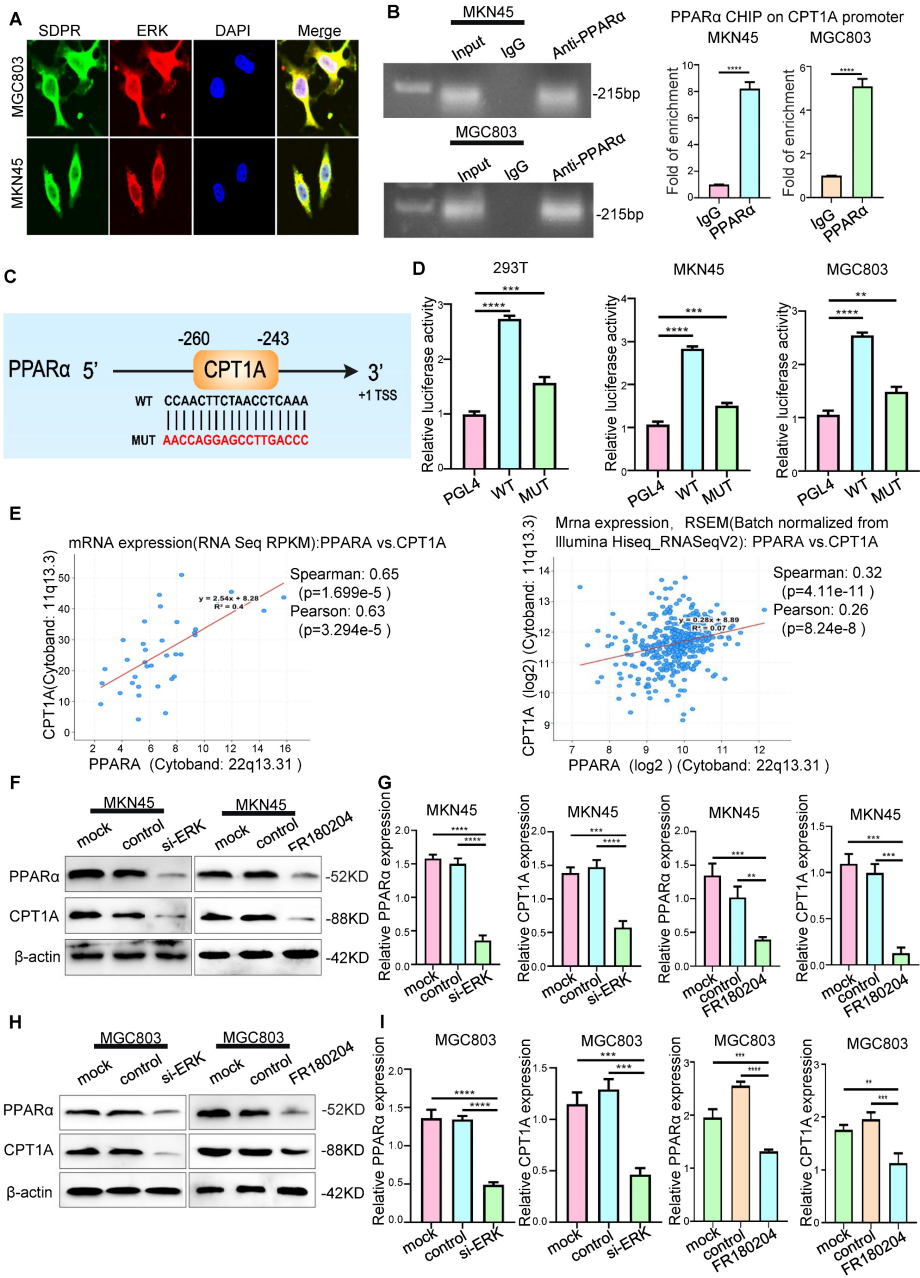
** SDPR regulates CPT1A through the ERK/PPARα pathway.** (A) Immunofluorescence microscopy colocalisation analysis of SDPR and ERK in MGC803 and MKN45 cells. Magnification, 180×. (B) After ChIP analysis using anti-PPARα antibody, PCR gels showed amplification of the PPARα binding site. (C) Schematic diagram of a potential pparα binding sequence in the CPT1A promoter region. (D) Relative luciferase activity of the indicated promoter vectors in 293T, MGC803, and MKN45 cells transfected with PPARα plasmids. (E) Comprehensive analysis of complex cancer genomic and clinical profiles using cBioPortal revealed that CPT1A expression was positively correlated with PPARα in two independent databases (TCGA Provisional and TCGA Panccancer Atlas). (F-G) Western blot and qRT-PCR were used to detect the changes in PPARα and CPT1A in MKN45 and MGC803 cells treated with ERK interference or inhibitor.** P < 0.01; *** P < 0.001; **** P < 0.0001. These experiments were repeated three times.

Next, the mechanism of SDPR regulating CPT1A was further explored. Our previous studies also confirmed that SDPR directly inhibits ERK phosphorylation by interacting with ERK at the protein level 9. Laser confocal microscopy revealed a high degree of co-localisation between SDPR and ERK, which further confirmed the protein-level interaction between them (Figure 5A). It has been reported that ERK promotes the expression of PPARα 21, 22. We confirmed by Western blot and qRT-PCR that ERK interference or ERK inhibitor (FR180204) can significantly downregulate the expression of PPARα and CPT1A (Figure 5F-I, S3A-B). Through analysis of transcription factor database JARSPAR, we found that there was PPAR binding site in the promoter region of CPT1A (Figure 5C). Meanwhile, studies have confirmed that PPARα regulates the expression of CPT1A and CPT1C at the transcriptional level, thereby promoting tumour FAO 23. Interestingly, integrative analysis of complex cancer genomics and clinical profiles using the cBioPortal 24, we found in two separate databases (TCGA Provisional and TCGA PanCancer Atlas) that the expression of CPT1A was positively correlated with PPARα (Figure 5E), further suggesting that PPARα may be a transcription factor of CPT1A in gastric cancer.

We further confirmed that PPARα protein was recruited to the binding site in the CPT1A promoter using ChIP assays (Figure 5B). A luciferase reporter assay was used to determine PPARα directly binds to CPT1A (Figure 5D). The results showed that PPARα upregulated the expression of CPT1A at the transcriptional level. Through KEGG enrichment analysis of differentially expressed genes in the microarray, we found that SDPR may regulate the PPAR signalling pathway (Figure 3A). These results further indicated that SDPR downregulated the expression of PPARα and CPT1A by inhibiting ERK. Therefore, we concluded that SDPR interacts with the ERK protein to inhibit CPT1A expression at the transcriptional level by regulating the transcription factor PPARα.

Finally, experiments were conducted to further clarify the regulatory effect of ERK on gastric cancer metastasis. After ERK interference or inhibitor treatment (Figure S4A-B), transwell and wound-healing experiments confirmed that cell invasion and metastasis were inhibited in MKN45 and MGC803 cells (Figure S4C-E).

### CPT1A promotes gastric cancer metastasis

**Figure 6:**
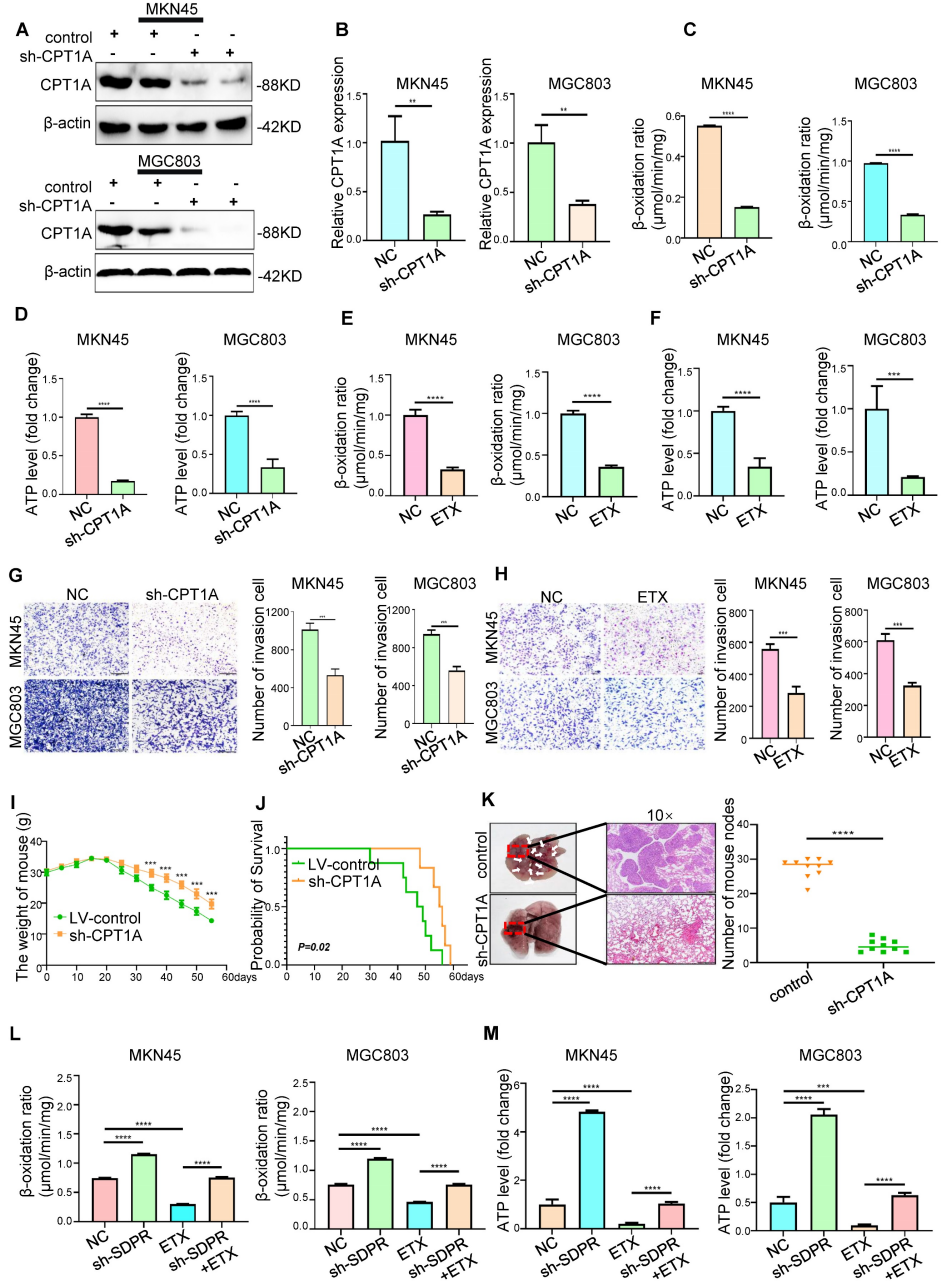
** CPT1A promotes gastric cancer metastasis and fatty acid oxidation.** (A-B) Western blot and qRT-PCR were used to verify the Knockout efficiency of CPT1A. (C-D) FAO rate and ATP level of MKN45 and MGC803 cells after CPT1A knockout. (E-F) FAO rate and ATP level of MKN45 and MGC803 cells treated with CPT1A inhibitor (ETX). (G) Transwell chamber migration assay was used to detect the migration and invasion ability of MKN45 and MGC803 cells after CPT1A knockout. (H) Transwell chamber migration assay was used to detect the migration and invasion ability of MKN45 and MGC803 cells treated with CPT1A inhibitor. (I) Body weight change curves of mice injected with CPT1A knockout cells and control cells. (J) Kaplan-Meier survival curves for the mice after injections with CPT1A knockout cells and control cells. *P* = 0.02. (K) Comparison of lung metastases in mice injected with CPT1A knockout cells and control cells. (L-M) The recovery experiment of the FAO rate and ATP level of MKN45 and MGC803 cells treated with ETX, sh-SDPR, or ETX+ sh-SDPR. ** P < 0.01; *** P < 0.001; **** P < 0.0001. These experiments were repeated three times.

Although previous gene microarray analysis and experimental results suggested that CPT1A is a downstream target gene of SDPR (Figure 4A), the potential clinical value of CPT1A in gastric cancer was still unclear. We found that CPT1A was highly expressed in most tumours according to TCGA microarray analysis (Figure S5A), suggesting that CPT1A may play a role in promoting tumours in gastric cancer. Etomoxir (ETX), an irreversible inhibitor of CPT1A, has been shown to inhibit FAO in a variety of tumours 25, 26. In MKN45 and MGC803 cells, CPT1A was inhibited by lentiviral knockdown or ETX, and downregulation of CPT1A was confirmed by Western blot (Figure 6A and Figure S5B; Figure S5E) and qRT-PCR (Figure 6B and Figure S5C). Transwell experiments (Figure 6G-H) and wound-healing experiments (Figure S5D; Figure S5F-H) confirmed that the downregulation or inhibition of CPT1A significantly inhibited the metastasis of gastric cancer. *In vivo*, compared with the control group, knockout of CPT1A significantly improved the survival and prognosis of nude mice (Figure 6I-J) and inhibited the formation of lung metastases (Figure 6K). HE staining of the tissue pathological sections of the model mice also confirmed that CPT1A had no significant effect on the heart, liver, spleen, or kidney (Figure S6A). The results of *in vivo* experiments further confirmed that CPT1A significantly promoted the metastasis of gastric cancer.

### Interference with lipid metabolism can inhibit gastric cancer metastasis

Gene function enrichment analysis of the GEO gastric cancer public database GSE57303 showed that CPT1A is associated with fatty acid metabolism and FAO (Figure 4D). Therefore, we explored the role of CPT1A in gastric cancer. In MKN45 and MGC803 cells, CPT1A knockout significantly inhibited FAO (Figure 6C) and ATP levels (Figure 6D). As a classic inhibitor of CPT1A, ETX could significantly inhibit the FAO rate (Figure 6E) and ATP level (Figure 6F) in MKN45 and MGC803 cells. In order to further verify that the metastasis of gastric cancer can be inhibited by interfering with lipid metabolism, we performed recovery experiments. SDPR knockdown significantly reversed the inhibitory effect of ETX on CPT1A (Figure S6B). SDPR knockdown significantly reversed the inhibition of FAO rate (Figure 6L) and ATP level (Figure 6M) by ETX. SDPR knockdown also significantly reversed the inhibitory effect of ETX on cell metastasis (Figure S6C). Therefore, interference with CPT1A, a key regulator of lipid metabolism, may inhibit gastric cancer transformation.

### SDPR/CPT1A is associated with clinical prognosis in gastric cancer

**Figure 7:**
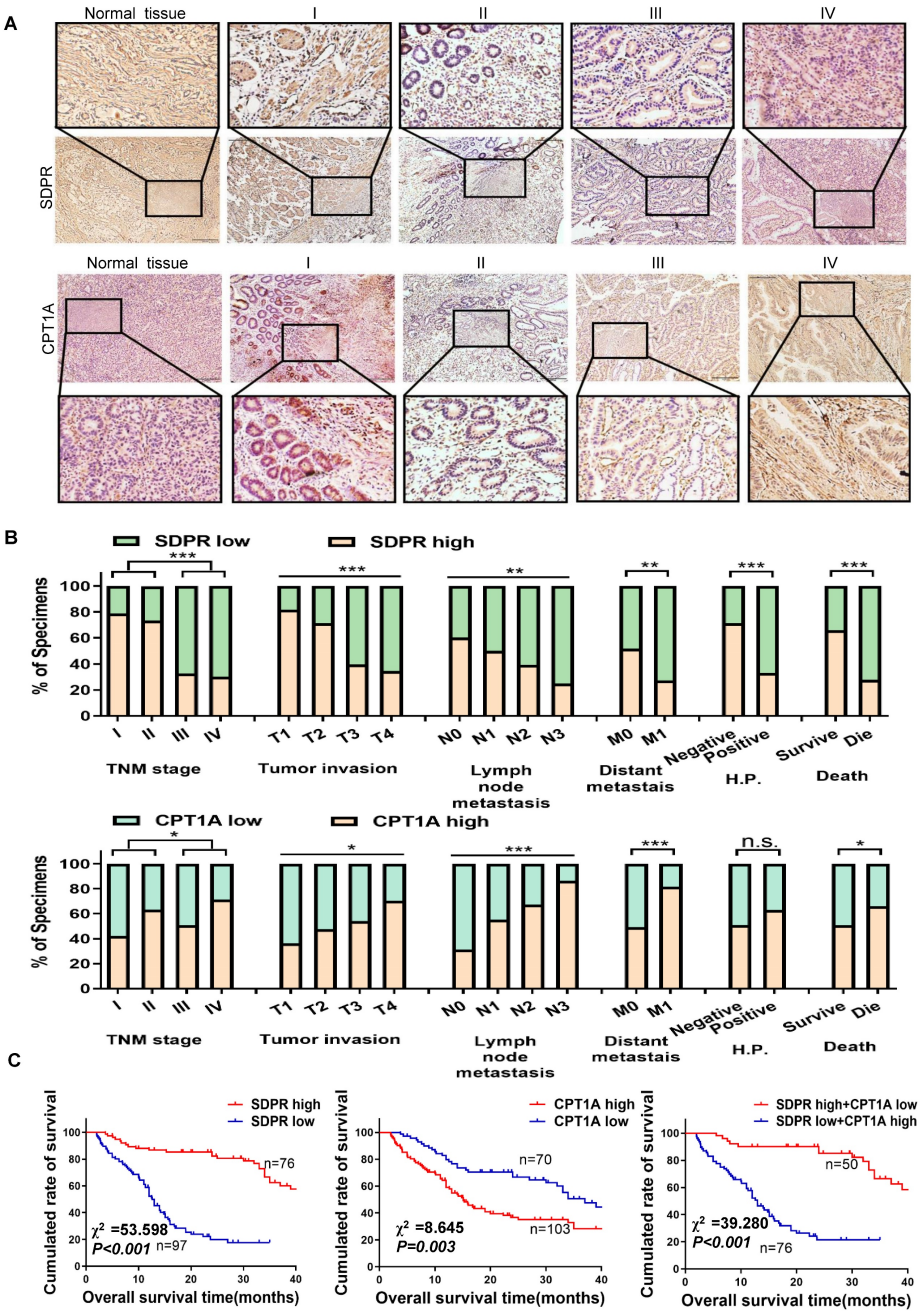
** SDPR/CPT1A is associated with clinical prognosis of gastric cancer.** (A)Immunohistochemical analysis was used to analyse the expression of SDPR and CPT1A in 170 normal human gastric tissues and gastric cancer samples of TNM stage I-IV patients. (B) Frequency of low and high SDPR/CPT1A expressions categorised by TNM stage, tumour invasion, lymph node metastasis, distant metastasis, HP positive, and death. Patients were separated into high- and/or low-expression groups by the expression score of the SDPR/CPT1A.(C) Kaplan-Meier plots of SDPR/CPT1A expression and overall survival were retrospectively analysed in 170 patients with gastric cancer.

The expression levels of SDPR and CPT1A were evaluated using immunohistochemistry analysis of 170 paraffin-embedded gastric cancer specimens and normal tissues. SDPR was expressed at low levels in gastric cancer, and its expression level decreased relative to the progression of the tumour stage, whereas CPT1A showed the opposite result (Figure 7A). The expression of SDPR and CPT1A was significantly correlated with TNM stage, distant metastasis, helicobacter pylori (HP)positivity, survival status, overall survival, and other clinical variables (Figure 7B). By analysing 170 clinical gastric cancer patients, we concluded that high expression of SDPR or low expression of CPT1A in gastric cancer patients was associated with better overall survival (OS), interestingly, patients with both high expression of SDPR and low expression of CPT1A had the best prognosis (Figure 7C).

Kaplan-Meier survival analysis showed that gastric cancer patients with high SDPR expression had better disease-free survival (DFS) and OS (Figure S7A), while those with high CPT1A expression had the opposite result (Figure S7B). The correlation between SDPR/CPT1A and clinicopathological features is shown in Table 1. Univariate survival analysis showed that high CPT1A expression (*P* < 0.001, hazard ratio [HR] = 1.817) and low SDPR expression (*P* < 0.001, HR = 0.195) were associated with shorter overall survival. In addition, multivariate survival analysis showed that SDPR and CPT1A expression, T grade, and age were independent predictors of prognosis in gastric cancer patients (Table 2).

## Discussion

We previously explored the role of TGF-β in gastric cancer EMT and tumour stemness and confirmed that SDPR is a downstream target of TGF-β 9. However, the role of SDPR in gastric cancer and its underlying mechanisms remained unclear. Previously, using gene chip analysis, we found that SDPR regulated lipid metabolism in gastric cancer. SDPR regulates the ERK/PPARα signalling axis by interacting with the ERK protein and regulates CPT1A at the transcriptional level, which is involved in FAO in gastric cancer. These results suggest that SDPR has a potential prognostic value and clinical significance in gastric cancer.

Lipid metabolism is an important metabolic pathway in the body that plays a crucial role in maintaining homeostasis in the intracellular environment 27. At the same time, lipid metabolism is vital for the maintenance of the malignant tumour microenvironment. Lipid metabolism produces ATP to supply energy to tumour cells, which further promotes tumour proliferation, migration, invasion, and other malignant biological behaviours 28. Jiang et al. reported that enhanced fatty acid metabolism promotes the invasive growth of glioblastoma multiforme through CD47-mediated immune evasion 29.

FAO is a process of lipid consumption, which can produce a large amount of energy for the function of tumour cells. In recent years, many studies reported that regulation of the key enzyme CPT1A in FAO affects the occurrence and development of various tumours, such as nasopharyngeal carcinoma and colorectal cancer. Tan et al. found that FAO was significantly active in radioresistant cell lines of nasopharyngeal carcinoma, CPT1A was highly expressed in radioresistant cell lines, and inhibition of CPT1A significantly improved the radiosensitivity of nasopharyngeal carcinoma 26. Wang et al. reported that CPT1A inhibits anoikis by promoting FAO and thereby mediating tumour metastasis 18. Based on the strong tumour-promoting effect of CPT1A, inhibitors and antagonists designed against CPT1A are potential anti-tumour strategies. ETX is an irreversible CPT1A inhibitor and *in vivo* and *in vitro* studies have shown that ETX inhibits the growth of prostate cancer tumours 30. This study confirmed that ETX can be used to inhibit tumour metastasis in gastric cancer. Therefore, targeted inhibitors of CPT1A may be a new strategy for the treatment of clinical tumours.

TGF-β is an important inflammatory factor in the tumour microenvironment. According to literature reports, high expression of TGF-β can mediate malignant biological behaviours, such as tumour proliferation, invasion, and drug resistance 31. Recent studies have shown that TGF-β1 aggravates the progression of cholangiocarcinoma cells *in vitro* and *in vivo* by activating the integrin beta-1 (ITGB1)-dependent PPARγ signalling pathway 32, and TGF-β upregulates lncRNA UCA1 to promote resistance of breast cancer cells to doxorubicin 33. We have also carried out studies on TGF-β and the malignant biology of tumours. On the one hand, TGF-β can regulate the expression of LASP1 and Mir-187, activate classic Smad2, and promote the digestive system tumour EMT 8. In contrast, TGF-β activates the non-Smad pathway by regulating the Mir-577 /SDPR signalling axis and promotes tumour stemness and EMT in the digestive system 9. This study further confirmed the negative regulation of SDPR by TGF-β and verified that TGF-β can promote the metastasis of gastric cancer by upregulating lipid metabolism. Therefore, further exploration of TGF-β is expected to provide a new direction for suppressing gastric cancer metastasis.

SDPR is a member of the fossa protein family, which are involved in biological processes such as endocytosis, lipid homeostasis, signal transduction, tumourigenesis, and development 34. Unozawa et al. demonstrated that low SDPR expression in oral squamous cell carcinoma can block the ERK pathway to inhibit tumour proliferation 35. Our study showed that SDPR is downregulated in gastric cancer, and this level is associated with cancer metastasis. Survival analysis showed that patients with high SDPR expression had a better prognosis, which was highly consistent with the findings in hepatocellular and thyroid carcinomas 10, 36. Recent studies reported that SDPR can induce apoptosis in hepatoma cells by activating the ASK1-JNK/p38 MAPK pathway 36. In breast cancer, SDPR can combine with phosphatidylserine (PS), inhibit ERK and NF- κB, and ultimately promote tumour apoptosis 37. Our previous studies identified that SDPR could interact with ERK at the protein level to inhibit its expression, which is consistent with the findings in breast and oral cancer 35. The ERK pathway plays an important role in various biological processes such as cell migration, proliferation, differentiation, and angiogenesis 38. In this study, through KEGG enrichment analysis of differential genes on the gene chip, we found that SDPR may regulate the PPARα signalling pathway and negatively regulate PPARα expression. Previous studies reported that ERK can regulate the PPARα pathway, and the results of luciferase reporter gene assays showed that PPARα can bind to CPT1A and regulate the expression of CPT1A at the transcriptional level. Based on our existing literature reports and our research results, it is clear that SDPR can act as a tumour suppressor gene in various tumours; therefore, it is expected to become a new target for suppressing tumour metastasis. At the same time, exploring the role of SDPR in other types of tumours and pathways will be the direction of our future studies.

In summary, overexpression of SDPR inhibits TGF-β-induced gastric cancer metastasis by inhibiting the key enzyme CPT1A of FAO, which plays an important role in regulating gastric cancer metastasis. Our study is the first to demonstrate that SDPR regulates lipid metabolism and serves as a bridge between TGF-β and lipid metabolism. The results of this study indicate that SDPR, as a tumour suppressor, plays a crucial role in inhibiting gastric cancer metastasis by regulating FAO processes, suggesting that SDPR is an important molecule connecting lipid metabolism and gastric cancer metastasis.

## Conclusion

In conclusion, TGF-β inhibits the expression of SDPR in gastric cancer, relieves the inhibitory effect of SDPR on the protein level of ERK, and promotes the expression of the transcription factor PPARα, thereby upregulating the expression of CPT1A at the transcriptional level, promoting FAO and inducing gastric cancer metastasis. This study provides a scientific basis for TGF-β/SDPR/CPT1A axis as a clinical therapeutic target for patients with gastric cancer.

## Supplementary Material

Supplementary figures and tables.

## Figures and Tables

**Table 1:** Correlation between SDPR/CPT1A and clinicopathological features

Characteristics		SDPR expression			CPT1A expression		
	n(%)	SDPR high	SDPR low	P-value	CPT1A high	CPT1A low	P-value
**Age (years)**							
**≥55**	98(56.65)	46	52		54	44	
**<55**	75(43.35)	30	45	0.440	49	36	0.766
**Gender**							
**Male**	92(53.18)	42	50		53	39	
**Female**	81(46.82)	44	47	0.768	50	31	0.642
**TNM stage**							
**I**	19(10.98)	15	4		8	11	
**II**	30(17.34)	22	8		19	11	
**III**	61(35.26)	20	41		31	30	
**IV**	63(36.42)	19	44	0.000	45	18	0.042
**Tumor invasion**							
**T1**	11(6.36)	9	2		4	7	
**T2**	21(12.14)	15	6		10	11	
**T3**	63(36.42)	25	38		34	29	
**T4**	78(45.08)	27	51	0.001	55	23	0.029
**Lymph node metastasis**							
**N0**	48(27.75)	29	19		15	33	
**N1**	38(21.97)	19	19		21	17	
**N2**	43(24.85)	17	26		29	14	
**N3**	44(25.43)	11	33	0.006	38	6	0.000
**Distant metastais**							
**M0**	118(68.21)	61	57		58	60	
**M1**	55(31.79)	15	40	0.003	45	10	0.000
**Tumor differentiation**							
**Well**	24(13.87)	17	7		15	9	
**Moderate**	59(34.10)	34	25		30	29	
**Poor**	90(52.03)	25	65	0.000	58	32	0.242
**Helicobacter pylori**							
**Negative**	49(28.32)	35	14		25	24	
**Positive**	124(71.68)	41	83	0.000	78	46	0.151
**Overall survival**							
**Survive**	73(42.20)	48	25		37	36	
**Die**	100(57.80)	28	72	0.000	66	34	0.043

**Table 2:** Univariate and multivariate analyses of individual parameters for correlations with overall survival rate: Cox proportional hazards model

Variables	Univariate				Multivariate		
	OR	CI (95%)	*P* value		OR	CI (95%)	*P* value
**Age (<55/≥55)**	0.545	0.434-0.759	0.042*		0.745	0.547-0.995	0.046*
**Gender (Male/Female)**	0.895	0.693-1.401	0.775		0.954	0.781-1.239	0.741
**T classification (III+IV/ I+II)**	1.982	2.041-7.585	0.000*		1.767	1.607-3.552	0.000*
**N classification(N1-3/N0)**	1.728	1.027-2.302	0.037*		1.863	0.953-2.176	0.083
**M classification(M1/M0)**	2.541	1.275-2.657	0.001*		1.853	0.979-2.285	0.062
**Differentiation (Poor / Well)**	1.529	0.488-1.640	0.		1.247	0.644-2.262	0.558
**Helicobacter pylori (+/-)**	1.456	0.614-1.949	0.101		1.130	0.905-3.085	0.101
**SDPR expression (High /Low)**	0.195	0.126-0.302	0.000*		0.213	1.607-3.552	0.000*
**CPT1A expression (High /Low)**	1.817	1.229-2.691	0.000*		2.012	1.607-3.552	0.000*

Abbreviations: OR, Odds ratio; CI, Confidence interval. * Statistically significant (P < 0.05).

## Abbreviations

TGF-β: transforming growth factor-β
SDPR: serum deprivation protein response
ERK: extracellular signal-related kinase
CPT1A: Carnitine palmitoyl transferase 1A
PPARα: peroxisome proliferator-activated receptor alpha
IHC: immunohistochemistry
EMT: epithelial-mesenchymal transition
LASP1: lim and sh3 protein 1
ChIP: Chromatin immunoprecipitation
HP: helicobacter pylori
HE: Haematoxylin and eosin
TCGA: The Cancer Genome Atlas
FAO: Fatty acid oxidation rate
DFS: disease-free survival
OS: overall survival
