# Supplementary material for: SDPR Inhibits TGF-β Induced Cancer Metastasis Through Fatty Acid Oxidation Regulation in Gastric Cancer
Source: Int J Biol Sci. 2023 Jun 4;19(10):2999–3014. doi: 10.7150/ijbs.83012 (PMC10321275; doi:10.7150/ijbs.83012)
Supplement: Supplementary file 1 — Supplementary figures and tables. [file ijbsv19p2999s1.pdf]

Supplement materials

Table S1. Sequence information used in this study

Name	Sequence
SDPR NC	5'- UUCUCCGAACGUGUCACGUTT -3'
SDPR siRNA1	5'-GGGACAACUCACAGGUGAATT-3'
SDPR siRNA2	5'-UCCUCCGACGCAACCAUUUTT-3'
SDPR siRNA3	5'-GCAGUGAGCAGAUGCCAAATT-3'
ERK NC	5'-UUCUCCGAACGUGUCACGUTT-3'
ERK siRNA	5'-GGACCUCAUGGAAACAGAUTT-3'
CPT1A NC	5'- UUCUCCGAACGUGUCACGUTT -3'
CPT1A siRNA	5'-GCCATGAAGCTCTTAGACAAA-3'

Table S2. The qPCR primers used for this study

Gene		Primer Sequence	GC(%)	Tm(℃)
SDPR	Forward	5'-CTCCGACGCAACCATTT-3'	52.94	55.09
	Reverse	5'-CTTTCTTGAGGCTATCCACTT-3'	42.86	55.30
PPARα	Forward	5'-TCGGCGAGGATAGTTCTGGAAGC-3'	56.52	64.20
	Reverse	5'-ACCACAGGATAAGTCACCGAGGAG-3'	54.17	63.47
ERK	Forward	5'-GTACCTGGCAGACATGAACTAT-3'	45.45	57.59
	Reverse	5'-GTATCGTCCTCTAGAAAGCGTG-3'	50.00	58.38
CPT1A	Forward	5'-CTCCGCCTGAGCCATGAAG-3'	63.16	60.52
	Reverse	5'-CACCAGTGATGATGCCATTCT-3'	47.62	58.35
β-actin	Forward	5'-CCTTCCTGGGCATGGAGTC-3'	63.16	63.16
	Reverse	5'-TGATCTTCATTGTGCTGGGTG-3'	47.62	58.56

Table S3. Information on antibodies used for this study

Antibody	Cat. No.	WB	IP	IF	IHC	Specificity	Source
SDPR	12339-1-AP	1:500			1:100	Rabbit	Proteintech Antibody
ERK1/2	11257-1-AP	1:1000				Rabbit	Proteintech Antibody
PPARα	ab227074	1:1000				Rabbit	Abcam Antibody
CPT1A	D12004	1:1000			1:100	Rabbit	Abmart Antibody
β-actin	BS6007M	1:5000				Mouse	Bioworld technology Antibody

Table S4. The expression profiling data of differential genes.

**Gene Name**	**Ave.control**	**Ave.SDPR**	**Fold change**
SDPR	112.0085217	13170.99801	117.7852045
CEMIP	947.51619	3535.845454	3.731083147
IL24	95.80579755	398.7677542	4.153651954
BOLA2-SMG1P6	95.16310436	330.0037202	3.461639403
TNFAIP6	381.9368028	934.6547021	2.446766828
CSF3	75.50718671	317.3308404	4.190705392
ASMTL	879.4409849	431.0923733	0.490081016
IL33	97.63741497	373.2892349	3.816960937

PRG4	1045.56454	2255.836132	2.157852608
CCL20	212.2997255	482.8542715	2.274383437
MMP1	14.06654191	73.85390478	5.199575112
STC1	749.6181528	2044.993409	2.726111487
RAC2	85.2724012	210.2823493	2.466690357
THBD	898.4711112	1842.282822	2.050539954
SAMSN1	372.6938234	806.3474241	2.163699503
CXCL8	359.5328252	921.4861452	2.561941658
IGFN1	76.84591265	222.7089868	2.893085502
C11orf96	103.6273498	238.9984838	2.308767107
S100A9	764.8853325	1571.466224	2.054378414
SSPO	243.6782992	552.7434827	2.267218361
MYEOV	126.1351488	321.6554302	2.547924408
IL11	79.13580344	181.8820397	2.291317721
EGR1	88.97797272	185.5078327	2.084586214
TMEM158	1723.068199	3509.822758	2.036611945
INHBA	33.97203341	141.3113802	4.106239209
ITGAX	169.80407	454.7532075	2.673727009
RHCG	205.4854416	459.1321593	2.231359211
SERPINA1	156.0753997	336.9232683	2.156598034
PTGS2	62.42970634	157.3018507	2.512953006
SPINK13	43.78913071	113.4419221	2.587979792
IL13RA2	7.765600032	37.55223793	4.766191465
ANXA10	33.15114408	95.78751413	2.872286538
SLX1B	32.21525769	264.983252	8.14047262
STAC2	65.38065819	25.92965945	0.397311504
RASD1	83.85950966	184.9943517	2.203940028
OSCAR	37.18010369	92.81733077	2.488549395
KRT5	71.0717401	31.26721716	0.4409586
C4A	50.36465781	111.2117486	2.202412121
SAMD15	40.02734733	14.1638969	0.35358446
HSD11B1	20.47273528	63.21987466	3.059160062
NLRP6	7.696846685	27.67678236	3.542197073
MYO15A	21.59619653	55.43844477	2.561079031
GJA1	17.93753108	51.27573227	2.850750911
BNIPL	22.38217419	55.6275675	2.472268808
RGS17	16.14670851	41.44764481	2.546695119
SRRM3	26.33225123	59.28884864	2.251908956
BOP1	136.6868414	39.98729625	0.288181867
CYP21A2	13.77825278	40.12391925	2.893858423
SCG5	49.95972967	112.3970954	2.241071699
MSMB	15.35190193	38.88235871	2.508435976
TREM1	25.07375157	72.00906081	2.846956023
CPEB1	5.222689524	20.1157688	3.762106635
GTPBP6	19.17473791	105.5787277	5.522142295
COL17A1	39.47722853	81.46786539	2.059934455
PKD1L2	27.60564287	58.1616305	2.095955847
TIE1	10.6768312	30.84301786	2.877754225
BHMT	15.18935073	3.589618037	0.247653264
CXCL1	13.21685507	34.67525289	2.619586888

COL6A3	5.698912175	24.90495513	4.21137328
ARHGEF35	8.622523984	25.46703146	2.94831249
AZIN2	28.36596318	59.86676609	2.104089524
SPINK6	60.94580549	158.0190294	2.556606976
DNER	29.63093731	61.97040789	2.085450448
RPE65	5.729070789	19.50998875	3.38402418
NTN5	18.7786611	44.61057175	2.369816015
PTPN22	6.385862483	21.25406751	3.294277757
ZNF497	61.40873293	30.74583284	0.499898972
RBM20	16.6651975	35.75159033	2.142598226
NID1	42.4784525	95.3516978	2.226197113
CDHR2	51.74934277	109.3642561	2.10257102
HEPHL1	51.57086258	114.1622658	2.194380556
S1PR1	14.94609426	33.67212936	2.252125829
FCGR3B	18.84324123	40.09298232	2.114540036
CILP	76.68417353	31.37253409	0.408675971
CCR7	15.81179721	5.021326726	0.320823422
TMEM132B	22.80893203	45.94696235	2.016444116
CYTH4	20.9227615	45.55133941	2.16960892
NOS3	7.983802753	20.71616147	2.54626075
ALPI	27.09208148	10.36395928	0.387420498
APCDD1L	17.70982841	40.16285234	2.248955376
ESRP2	13.44048923	29.90044121	2.213059939
GIMAP2	13.26881896	3.203265668	0.244363403
LGALS9C	16.26724963	36.10396602	2.198069408
MRO	46.23234283	22.11243566	0.478583158
CSF2	6.00660953	21.18966085	3.442666667
CORO2B	31.77744711	63.78040742	2.003928944
EIF3CL	126.9127645	422.8651182	3.290977064
CDHR3	7.692970647	1.011656872	0.136820652
CCL3	12.56282556	28.38639891	2.239881376
VANGL2	27.9885532	11.90358506	0.426835362
VGF	45.06050586	96.24631881	2.11922558
ACOX2	19.4120861	45.73771133	2.335222996
GTF2IRD2B	47.18093337	96.00557919	2.035025119
EIF3C	1536.422822	615.6995273	0.401438672
SERF1A	7.414638846	19.1735532	2.558261926
RGPD6	137.2793082	302.9010097	2.218930706
C2orf66	8.164968644	19.89641057	2.429332932
SMIM24	8.771677175	1.935766918	0.225345796
C3AR1	6.633015136	16.82444771	2.49349924
RPS17	76.70073087	181.2310274	2.330956541
DNAI1	21.20796477	8.530587796	0.403257564
SMN1	677.5742267	1365.476865	2.012090084
PCDHGA4	6.968234869	1.130849585	0.187067301
PHKG1	15.44745536	32.91458109	2.129885863
EXOC3L1	11.71821741	29.2909483	2.473737558
ATG9B	7.572966011	21.04232882	2.719641448
TCAP	16.11659244	5.440469957	0.345234126
CHGB	8.08931705	23.52084718	2.842923307

KLRG2	8.925944292	20.45202333	2.28729153
CAGE1	13.10703926	1.888299633	0.151621299
OVOL1	9.095821083	20.32910968	2.213425131
SERPINB2	17.20583634	53.03048616	3.001381733
PTH1R	23.36815475	11.45332423	0.494210645
NTSR1	8.194798831	19.58632052	2.380356504
TIAF1	19.95487615	8.493603681	0.432418908
SCNN1G	6.678777627	1.224609539	0.200353691
SERF1B	40.02790641	87.70086975	2.210386269
GTF2IRD2	27.14312179	74.96595584	2.696670029
NOVA2	10.72630624	22.98916481	2.138980151
ZFPM2	10.159351	3.187767715	0.317301445
CALB2	14.64388993	4.773838112	0.330124334
LRRC4B	11.83347277	25.00194695	2.090698677
FOXD4	7.158566811	1.363040045	0.1909987
SLC25A6	755.2945248	193.8998524	0.259852185
IL3RA	10.99188206	26.97993784	2.45245934
SLC22A20	13.09326834	3.325238289	0.263331037
SERPINC1	6.054537327	1.205121829	0.22004735
AKR1B10	49.41300151	22.67220366	0.461528805
TFF2	7.530594456	1.130849585	0.171130894
CRAT	6564.591463	1892.797686	0.288334422
PRRG2	11.25367036	3.024317101	0.279324471
SLCO1B1	5.626315375	13.7026698	2.399421385
FITM1	5.647576616	1.179272818	0.233730575
AASS	6.145003207	16.10440832	2.607412873
KLHL6	8.086873124	1.370455272	0.171933112
HSPA6	29.66096749	71.37192379	2.346311831
FAM71E1	7.921099384	19.3654196	2.405995291
FAM186A	11.52551178	3.9592339	0.353139362
LMOD1	11.9394319	24.84343504	2.070444948
CCDC87	12.20837043	3.684624219	0.30864861
KIR3DL3	15.66942808	5.808564405	0.370984047
CDH5	7.681851406	1.888299633	0.256084715
C20orf96	6.851067841	16.97657995	2.424837156
CLEC18B	11.36854456	25.17465524	2.18457209
ECSCR	5.704556622	13.56753742	2.361786976
WNT2B	22.59594912	47.24012195	2.085051386
UBL4B	10.41052499	22.94501999	2.171459947
SLC17A7	7.8401641	16.41323574	2.091083533
BIRC7	6.764447335	15.21730137	2.244612783
LRRC15	9.833762815	20.98199818	2.113854806
GPR182	6.540563364	1.451793657	0.244382923
HBE1	6.495272858	1.224609539	0.204232886
ELOVL2	6.217246409	1.224609539	0.214081354
TMC4	9.58873312	20.17444332	2.096209053
C17orf105	5.817967952	1.031439085	0.180791158
MPIG6B	7.728002763	16.96648729	2.173116955
TRIM31	11.69610587	4.79265108	0.411002457
FAT2	22.29543825	9.905117094	0.445641598

SHH	7.659966539	16.42887332	2.118887476
SHD	15.66942808	5.705073472	0.36760045
CAMK1G	5.028630882	15.40703461	2.958171096
CCL3L1	7.936699605	18.89060324	2.352774094
VWA1	7.910596678	15.79809981	2.001792731
PLAC4	5.111452218	15.7361262	2.979875821
SHANK1	8.44752986	19.70668586	2.288282346
GRIN3B	8.555549868	17.13157991	2.002788371
DHH	5.903774031	15.0254752	2.514262006
KCNC1	6.757484274	1.884451646	0.290829695
CAMK2A	6.270883935	15.36279975	2.402771577
CAMK2B	6.85972545	15.76277807	2.279962821
DNALI1	7.429123274	2.365559755	0.337633375
WNT7A	5.549208374	13.13101878	2.323336492
LRRC34	7.10124255	14.2572026	2.000550271
RP1	6.9460934	16.07500452	2.278564728
TMEM89	7.869761984	2.643605985	0.354598316
TCN2	6.689135863	13.64205119	2.019746849
ARPC4-TTLL3	6.338939669	13.34506519	2.084936005
GNGT1	12.37267476	5.783069137	0.476957014
KLRC3	12.89749413	6.107052221	0.481195277
SPEF1	11.87010981	5.556108462	0.474365784
UBASH3A	7.209161874	1.715488603	0.237336698
CELF3	7.105353386	14.42722982	2.017750632
PIWIL2	7.455895816	15.69021573	2.092425465
PPP5D1	11.39658683	3.923902035	0.352711012
GOLGA6B	7.638146891	17.93650997	2.29735186
CKMT1A	8.997990028	3.753191618	0.422594621
PRKCG	6.938951774	14.50110311	2.056745409
CCDC85A	12.72630005	6.221020042	0.493980076
CACNA1B	12.48192165	6.107052221	0.498165237
ALS2CR12	11.37278826	5.450639999	0.490100353
SMIM2	10.28083833	4.990773486	0.491647871
KCNS2	6.367837873	1.772783053	0.29726337
C12orf80	6.740987406	2.417188175	0.376700766
C11orf53	6.297618618	14.02975753	2.185128307
ACOXL	13.3199938	6.405374208	0.481223489
CABYR	10.8989343	5.105277861	0.481216041
GPR35	8.594766656	19.00102215	2.205080577
PI15	7.303278024	2.591098673	0.372212171
FAM106B	5.028630882	12.2453346	2.378149399
HIST4H4	9.920578558	3.99399453	0.412807189
PPARA	12092.67235	2340.025371	0.193507713
CRYGN	14.47340376	7.076130819	0.494199739
MTRNR2L8	6.45741136	13.6259586	2.096946488
XAF1	5.375884601	1.811206988	0.359155099
SLC5A9	11.8930918	5.218961409	0.438822235
PRLR	5.014431763	10.3318821	2.057230771
ASGR2	6.060766644	1.250238711	0.222710248
HIST2H3D	9.222735865	3.861152422	0.421043245

SLC6A15	11.44116103	23.45681523	2.033561749
MANSC4	6.572345663	2.417188175	0.38395934
TMEM145	5.222689524	10.69648756	2.016954303
RGN	8.649051733	3.434283089	0.405335268
NAALADL2	5.634982948	1.370455272	0.247382827
HIST1H2BG	8.027536788	3.6593224	0.467195574
SMC1B	16.39019484	8.0386219	0.496670864
GSG1L2	8.987128247	4.317114376	0.48686721
IL37	9.857461828	4.234511878	0.439872686
KRT23	8.997990028	3.93704549	0.437051757
CDKL2	10.86052629	4.724515901	0.444323647
SLC26A8	5.053863415	1.811206988	0.38061368
PCDHB7	6.049232265	2.365559755	0.412756413
GPR34	5.310904311	2.080072985	0.415066951
RAD21L1	6.002146932	12.86958386	2.13058928
SYNDIG1L	10.08388499	3.784078615	0.368050103
ANKRD2	9.992615979	4.12884672	0.405539669
IFT74	16.14600408	7.352750356	0.445935355
FAM47E	5.056297792	1.544079671	0.319904122
SLC9C2	6.769147326	2.505214082	0.381789581
LGSN	8.954829207	3.844324476	0.434382877
NELL2	5.768299151	2.454724208	0.440126881
RAB17	10.45987857	5.10774048	0.489111207
HIGD1B	5.183197057	1.941214464	0.381032662
P2RY4	6.470326481	2.785617091	0.441184209
APOC3	6.951475692	3.108022129	0.453404983
LIPI	12.81006693	6.254915516	0.489976104
GKN2	5.290440103	2.365559755	0.46952828
CNR1	7.390172788	3.215202286	0.442130335
NIM1K	5.40468333	2.205022637	0.419565196
FBXL22	6.50862479	3.064289794	0.482260217
AEBP1	5.698912175	12.8239579	2.175269967
PCYT1B	6.174829533	2.409829151	0.389849702
THEGL	5.176580926	1.701039205	0.345287697
KCNJ4	5.581248916	2.270594561	0.417106775
CRLF2	30.61700991	64.93552942	2.14703231
ODF3L2	5.626315375	2.412606221	0.425913156
SPINK5	8.398830408	3.911867919	0.476467896
PALM3	7.283465306	3.592955308	0.498400099
LRRC17	5.250059759	2.365559755	0.463663428
C10orf107	5.192938086	2.443645078	0.477812872
PRCD	5.336371634	2.443645078	0.47039177
SIT1	5.089078533	1.985893184	0.400328539
HMSD	5.581248916	2.714468587	0.482422903
POPDC2	5.546696973	2.546109466	0.46555452
PSAPL1	5.40832345	2.532276674	0.474922477
RAB40AL	5.42989858	2.405111755	0.4396846
C1orf210	6.340492335	2.88551408	0.471273428
CBY3	6.060766644	2.831669747	0.481601856
CPT1A	13600.66024	3434.751154	0.252542972

ACOT8	1424.945367	454.2669628	0.318796056
AKT2	6414.568364	2951.131669	0.460067069
ABCD1	7679.049057	2696.074326	0.351094817
AKT1	18366.62257	8346.299661	0.45442757

Supplementary figures

Figure S1

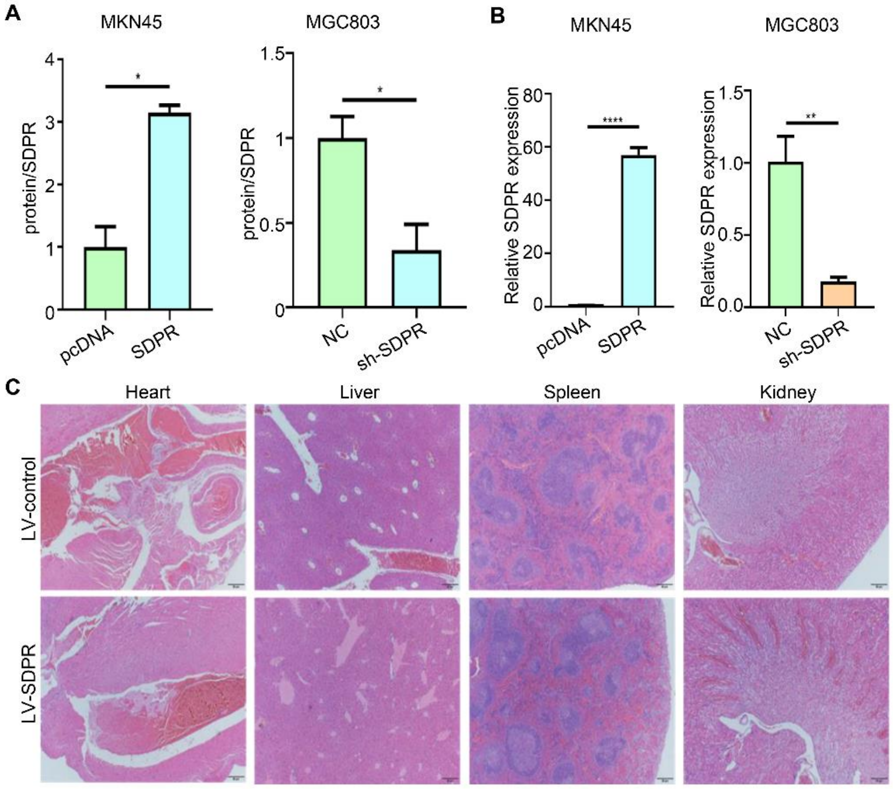

Figure S1 SDPR inhibits tumour metastasis in vitro and in vivo

(A) Western blot was used to verify SDPR overexpression and knockdown efficiency. (B) qRT-PCR was used to verify SDPR overexpression and knockdown efficiency. (C)HE staining of histopathological sections of heart, liver, spleen and kidney of model mice. ** $P < 0.01$; *** $P < 0.001$; **** $P < 0.0001$

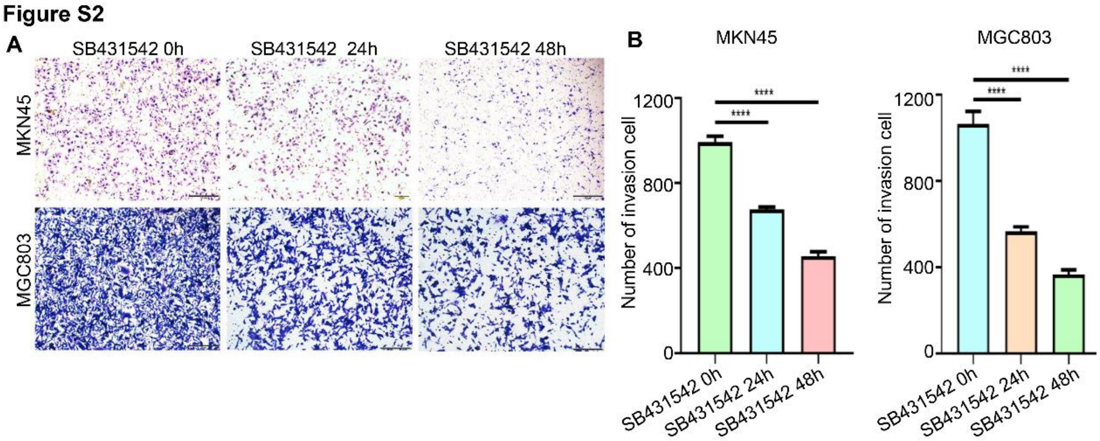

Figure S2 SDPR is involved in TGF-β-mediated gastric cancer metastasis

(A-B) Transwell chamber migration assay was used to detect the migration and invasion ability of MKN45 and MGC803 cells treated with TGF-β inhibitor for 0, 24, or 48h. The cells were counted under a microscope in five randomly selected fields. ** P < 0.01; *** P < 0.001; **** P < 0.0001. Magnification, 100×.

Figure S3

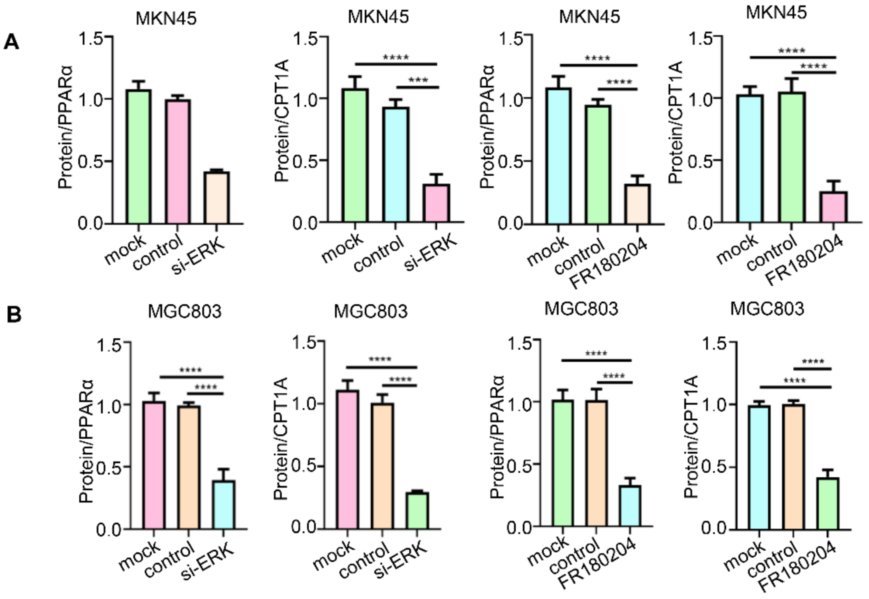

Figure S3 SDPR regulates CPT1A

(A)Western blot was used to detect the changes in PPARα and CPT1A in MKN45 cells treated with ERK interference or inhibitor. (B) Western blot was used to detect the changes in PPARα and CPT1A in MGC803 cells treated with ERK interference or inhibitor. ** $P < 0.01$; *** $P < 0.001$; **** $P < 0.0001$

Figure S4

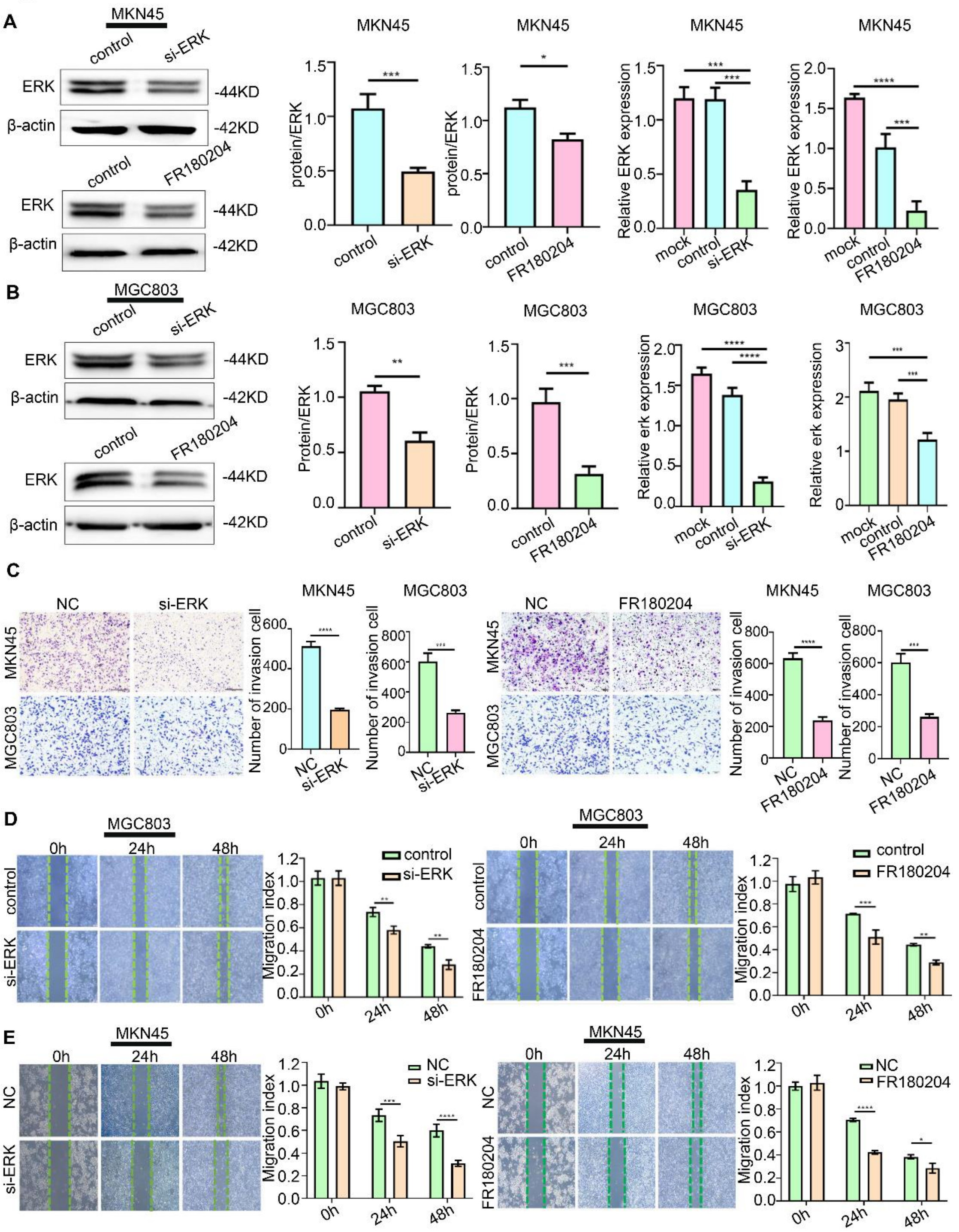

Figure S4 SDPR regulates CPT1A through the ERK/PPARα pathway

(A)Western blot and qRT-PCR were performed to verify the efficiency of ERK interference or suppression in MKN45 cells. (B) Western blot and qRT-PCR were performed to verify the efficiency of ERK interference or suppression in MGC803 cells. (C) Transwell chamber migration assay was used

to detect the migration and invasion abilities of MKN45 and MGC803 cells after ERK interference or suppression.(D) A wound healing assay was used to detect the migration ability of MGC803 cells after ERK interference or suppression. (E) A wound healing assays were used to detect the migration ability of MKN45 cells after ERK interference or suppression. ** P < 0.01; *** P < 0.001; **** P < 0.0001

Figure S5

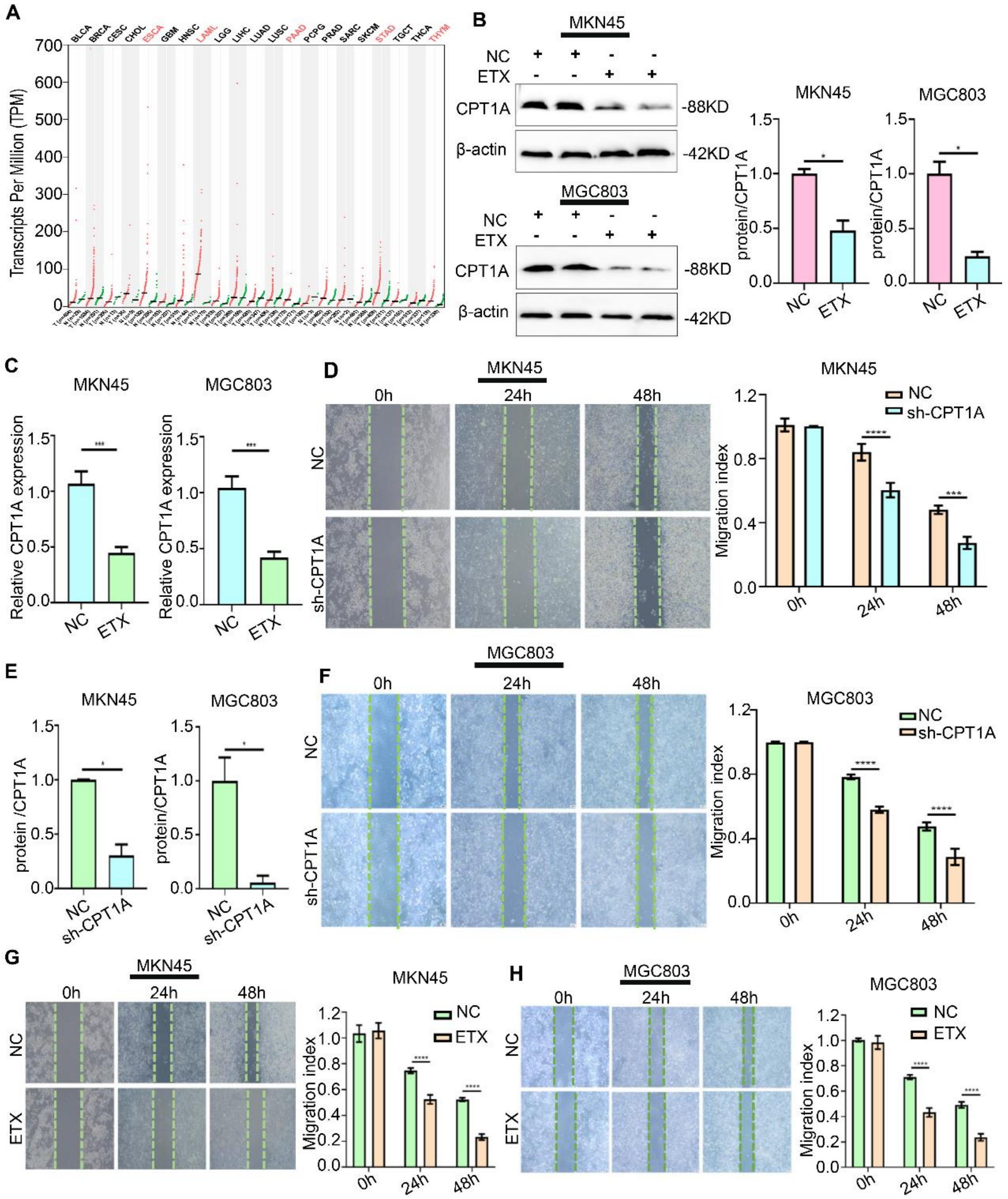

Figure S5 CPT1A promotes gastric cancer metastasis

(A)CPT1A was highly expressed in most tumours according to TCGA microarray analysis;(B-C) Western blot and qRT-PCR were used to verify the inhibition efficiency of CPT1A in MKN45 and MGC803 cells treated with ETX. (D) Wound healing assay were used to detect the migration ability of MKN45 cell after CPT1A knockout. (E)Western blot was used to detect the knockout efficiency of

CPT1A in MKN45 and MGC803 cells. (F-H) Wound healing assay were used to detect the migration ability of MGC803 and MKN45 cells after CPT1A knockout or suppression. * $P < 0.05$; *** $P < 0.001$; **** $P < 0.0001$

Figure S6

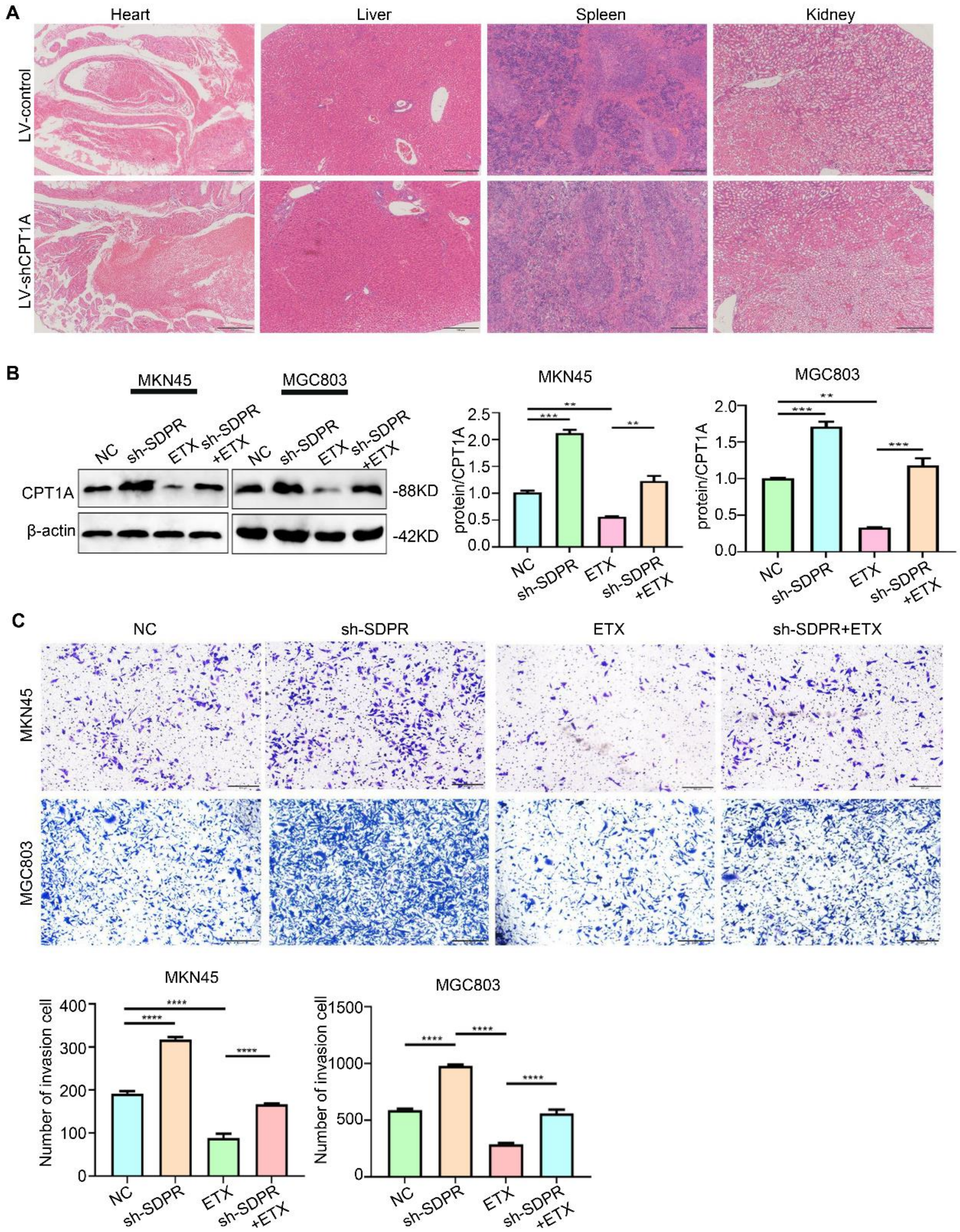

Figure S6 CPT1A promotes gastric cancer metastasis and fatty acid oxidation

(A) HE staining of histopathological sections of heart, liver, spleen and kidney of model mice. (B) Western blot recovery assay confirmed that knockdown of SDPR could restore the inhibitory effect of ETX on CPT1A. (C) Transwell recovery assay was used to detect the migration and invasion ability of MKN45 and MGC803 cells treated with ETX、sh-SDPR、 or ETX+ sh-SDPR. ** $P < 0.01$； *** $P <$

0.001； **** $P < 0.0001$

Figure S7

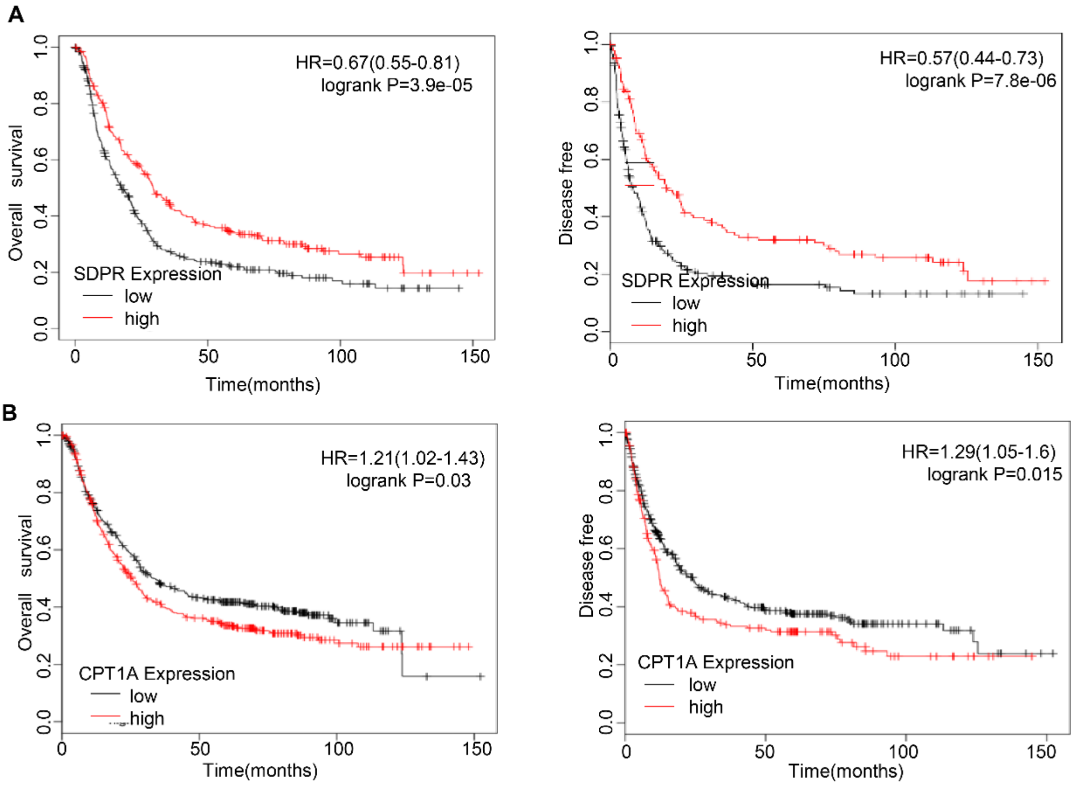

Figure S7 SDPR/CPT1A is associated with clinical prognosis in gastric cancer

(A-B) Kaplan-meier survival analysis was performed to analyze the relationship between SDPR and CPT1A expression and the prognosis of patients by database analysis. * $P < 0.05$; ** $P < 0.01$; *** $P < 0.001$; **** $P < 0.0001$
